# Persistent Homology Combined with Machine Learning for Social Network Activity Analysis

**DOI:** 10.3390/e27010019

**Published:** 2024-12-30

**Authors:** Zhijian Zhang, Yuqing Sun, Yayun Liu, Lin Jiang, Zhengmi Li

**Affiliations:** 1 Faculty of Science, Kunming University of Science and Technology, Kunming 650500, China; zhijian@kust.edu.cn (Z.Z.); 15924781512@163.com (Y.S.); tojianglin@163.com (L.J.); 15284696912@163.com (Z.L.); 2 Research Center for Mathematics and Interdisciplinary Sciences, Kunming University of Science and Technology, Kunming 650500, China

**Keywords:** social networks, persistent homology, machine learning, persistent entropy, clustering

## Abstract

Currently, the rapid development of social media enables people to communicate more and more frequently in the network. Classifying user activities in social networks helps to better understand user behavior in social networks. This paper first creates an ego network for each user, encodes the higher-order topological features of the ego network as persistence diagrams using persistence homology, and computes the persistence entropy. Then, based on the persistence entropy, this paper defines the Norm Entropy-NE(X) to represent the complexity of the topological features of the ego network, a larger NE(X) indicates a higher topological complexity, i.e., the higher the activity of the nodes, thus indicating the degree of activity of the nodes. The paper uses the extracted set of feature vectors to train the machine learning model to classify the users in the social network. Numerical experiments are conducted to evaluate the performance of clustering quality metrics such as profile coefficients. The results show that the proposed algorithm can effectively classify social network users into different groups, which provides a good foundation for further research and application.

## 1. Introduction

The growth of online social networks has provided users with a fascinating platform to share their knowledge and interests [1]. In social networks, users can be divided into different types according to their activity levels [2]. These different types of users together constitute a diverse community, and their activities and interactions affect the development and evolution of social networks. Users can be classified into different categories based on their activity levels: Active users, these are the most active and influential group of users in social networks. They frequently post content, participate in discussions, share information, and regularly interact with other users. Average users, these are the most prevalent group in a social network. They may occasionally post content, like, comment, or share information. Potential users, these are registered for an account but seldom or never participate in social network activities.

Currently, methods for classifying user activity in social networks are mainly based on network centrality metrics, for example, classifying the activity level of a user. However, most of them do not consider the high-dimensional topological information of the network, and the persistent homology theory can capture the high-dimensional topological structure of the network, so in this paper, we consider using persistent homology to classify active and inactive users in social networks.

Considering that there is some diversity in the behavior of social network users. This paper classifies users from two classes to multiple classes according to the average degree of active users in each class until the average degree of active users in the class is maximum. Choosing topological features with different dimensions also affects the classification results. In this paper, the persistence entropy of combining zero, one, and two dimensions and the persistence entropy of combining only one and two dimensions are chosen for comparison. The method in this paper is divided into three steps: in the first step, k-hop ego networks are built for each user. In the second step, the persistence homology of each ego network is calculated, and the persistence entropy of different dimensions is calculated. Based on the persistence entropy, the normative entropy-NE(X) is defined, which is used to analyze the topological complexity of each ego network, the higher the topological complexity, the more active the central node of the ego network. The third step combines NE(X) and machine learning to obtain the classification of the user in the social network.

## 2. Related Work

This chapter will discuss work related to the analysis of user activity on social networks and persistent homology.

### 2.1. Analysis of User Activity

The large number of active users in social networks provides valuable data and scenarios for the study of network behavior, data security, information dissemination, and other interdisciplinary issues [2], and thus user activity has always been the focus of social networks [3,4,5]. He Chanyang [6] et al. analyzed the reasons why social networks become inactive from the perspectives of user-generated and consumed feeds, and by analyzing the content of user-generated feeds, they proposed a new perspective to understand social networks, i.e., exploring the change in user activity of this community from the content producer and consumer models. Shi Lei [7] et al. studied the characteristics of different types of users in microblogging services and proposed a method to use appropriate weights for different types of users to comprehensively calculate the user’s activity index. In the experiments, it was shown that using this method to calculate the active index of users in microblogging services can not only better reflect the ranking of users in the system but also show the overall distribution trend of other indicators. Ran Xiaobin [8] and others explore the influencing factors of users’ active behavior from the perspective of network externality, expanding the research on users’ continuous use behavior in online social networks and the way of measuring network externality, focusing on the differences in the contribution of different nodes to network externality, which is of some significance for future research on network externality.

### 2.2. Persistent Homology Theory

Persistent homology is one of the main tools for topological data analysis and is an effective method for portraying the topological features of a dataset. Persistent homology can give information about the shape of subgraphs, as well as the homology invariants of graphs, by identifying topological features that persist at multiple scales [9]. Topology complements standard feature representations, making persistent homology an attractive feature extractor for machine learning [10].

The core idea of persistent homology is to use different filters to transform the data. When applying persistent homology, first construct simplex complexes to transform the data into simplexes. Secondly, the topological structure of the data is analyzed by computing topological invariants (such as the Betti numbers). Finally, the calculated topological invariants can be formed into persistence diagrams or barcodes. By analyzing the points in a persistence diagram or the barcodes in a persistence barcode, the topological features of the data that have a long duration can be identified [11], which can be regarded as the salient structures, i.e., the stable structures in the data. Persistent homology has been used in some fields such as, for example, image segmentation [12], weighted persistent homology [13,14], and collaborative networks [15].

Currently, there is a gradual increase in research on the application of persistent homology in social networks. Claire Songhyun Lee [16] and others proposed the use of persistent homology to represent online social network graphs, which provides a method for analyzing network graphs without exposing sensitive information by converting them to the persistent homology barcode format. The method can accurately capture network topological features. Zhong Hui et al. [17] proposed a key node discovery method based on persistent homology. In their paper, they defined a node simplex centrality based on persistent homology theory and introduced a new metric for describing node importance in online social networks. They also proposed a key node discovery algorithm based on simplex centrality to obtain the importance ranking of nodes in social networks. Later, the authors used persistent homology to partition communities [18]. Bernadette J. Stolz [19] and others used topological data analysis to study “functional networks” constructed from time-series data from experimental and synthetic sources and found that persistent homology can detect differences in synchrony patterns in a dataset over time, thus providing insight into changes in the structure of communities in the network.

## 3. Combining Persistent Homology and Machine Learning to Classify Users by Activity Level

This Section describes the main methodology used in this paper. Section 3.1 shows how to create an ego network for each user. In Section 3.2, the difference between active and inactive users is explained after calculating the persistent homology of the ego network and visualizing it as a persistence diagram and barcodes. In Section 3.3, the classification method combining persistent homology and machine learning is explained. The main methodology of this paper is as follows:

Social network-G(V,E);

(1) Create a per-node ego network $G_{i}(i=1,2,\ldots ,n)$;
(2) Calculate the persistent homology of each ego network;
(3) Visualize as persistence diagrams-$PD_{i}$;
(4) Calculate the persistence entropy for the different dimensions of each $PD_{i}$;
(5) Define NE(X) to describe the user’s activity level;
(6) Combining NE(X) with machine learning to categorize users by activity level.

### 3.1. Create a Per-User Ego Network

In this paper, we study social networks where vertices represent users and edges represent relationships between users. We first create an ego-network [20] for each user. The nodes of each ego-network network consist of a unique central node, and the neighbors of this node, and the edges consist only of edges between the central node and its neighbors, and between neighbors and neighbors. It can therefore be used as a different feature for each observation. This is the key network used in this paper to classify actives from inactive.

**Figure 1:**
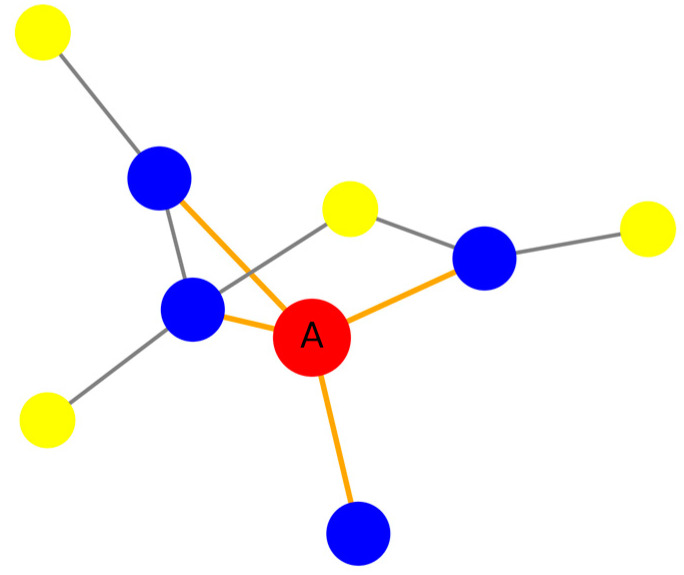
The Ego-network of A.

For example, in a social network, for a specific user A, we consider this user and their neighbors as nodes. By only considering the connecting edges between this user and their neighbors, we obtain an ego network centered around A, as illustrated in Figure 1.

In Figure 1, the value of k represents the range of the network, indicating how many hierarchical levels of neighbor nodes are included. This paper considers the high-dimensional topological structure of social networks; therefore, we chose ego-centered networks with k = 1 and k = 2. That is, the ego network of A includes node A and its two layers of neighboring nodes, along with the connections between them.

### 3.2. Persistent Homology in the Ego Network

After obtaining the egocentric network of each node, the persistent homology of each egocentric network is computed to extract the topological features of the ego network, and finally, the features are visualized as persistence diagrams and persistence barcodes. The process is as follows.

(1) Construct Simplicial Complex:

Convert the ego-network into a series of Rips complexes. Rips complexes capture the topological structure of the network by adding higher-order simplices (such as triangles).

For example, the simplicial complex constructed from the self-network in Figure 1 is represented as ([0, 1], 1.0), ([1, 2], 1.0), ([2, 3], 1.0), ([4, 5], 1.0), ([5, 6], 1.0), ([6, 7], 1.0), ([7, 8], 1.0), ([0, 2], 2.0), ([1, 3], 2.0), ([3, 4], 2.0), ([4, 6], 2.0), ([5, 7], 2.0), ([6, 8], 2.0),([0], 0.0), ([1], 0.0), ([2], 0.0), ([3], 0.0), ([4], 0.0), ([5], 0.0), ([6], 0.0), ([7], 0.0), ([8], 0.0).

(2) Compute Persistent Homology:

For each simplicial complex, compute its corresponding homology groups. Homology groups describe the connectivity and cyclic nature of the topological space. Persistent homology focuses on how these homology groups change over time (i.e., as the simplicial complex grows).

**Figure 2:**
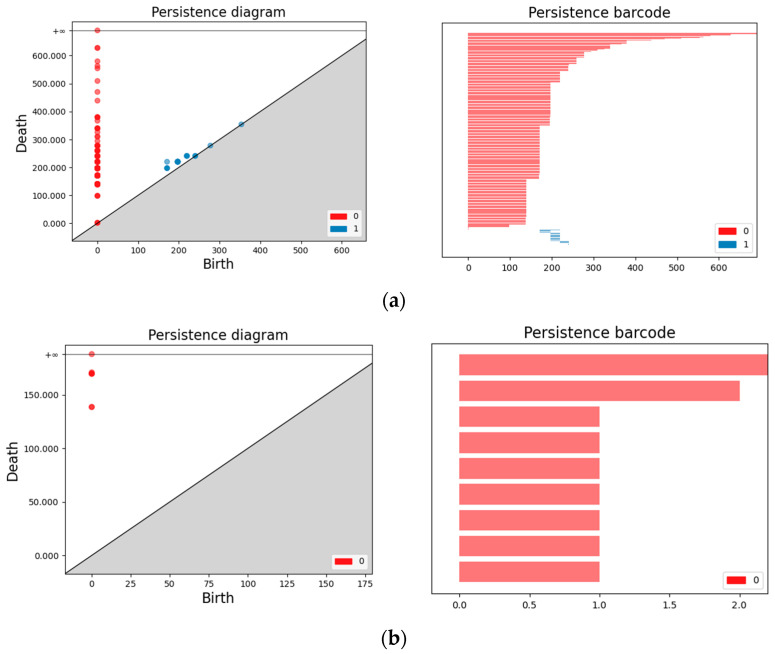
Persistence diagram and barcode for active (**a**), and persistence diagram and barcode for inactive (**b**).

(3) Generate Persistence Diagrams and Barcodes:

Persistence diagrams and barcodes are two methods for visualizing the results of persistent homology. The persistence diagram and persistence barcode for the previously mentioned self-network output are shown in Figure 2b.

Persistence Diagram: Each point represents a homology class. The horizontal axis indicates the birth time (when the class first appears in a simplicial complex), and the vertical axis indicates the death time (when the class disappears). Homology classes with longer lifespans appear closer to the diagonal in the diagram.

Persistence Barcodes: Each bar represents a homology class. The horizontal axis indicates the time range (birth to death), and the vertical axis indicates the index of the homology class. Longer bars correspond to homology classes with longer lifespans.

Since egocentric networks formed by active individuals have a different topology than egocentric networks formed by inactive individuals, and active individuals have more activities in the network compared to inactive individuals, active users therefore have more persistent features and more high-dimensional features, which means that the persistence diagrams and barcodes of the two are different, and the following Figure 2 shows the persistence diagrams and barcodes of active and inactive users.

In Figure 2, the red dots and red barcodes are 0-dimensional persistent features, and the blue dots and blue barcodes are 1-dimensional persistent features. 0-dimensional features usually refer to connected components, whereas 1-dimensional features refer to cycles. Users with longer feature durations and more high-dimensional features in Figure 2a are active users, while users with shorter feature durations and fewer high-dimensional features in Figure 2b are inactive users. This means that active and inactive users can be observed from the duration graphs and barcodes.

Therefore, we use the complexity of topological features to describe the user’s activity. If the topology feature is more complex, the more active the user is.

### 3.3. Categorizing Users by Activity

The persistence features obtained from the ego network cannot be directly input into machine learning for classification, so this paper considers processing the persistence feature data to obtain the persistence entropy [21], and then input them into machine learning for classification. Persistent entropy is an important concept in persistent homology analysis. Persistent entropy can be defined as the information entropy of the probability distribution of persistence intervals in the persistence diagram. Persistence intervals are the duration periods of topological features during topological decomposition, and they can be used to represent the evolution and change of different topological features in a dataset.

Let $\;X=\left\{I_{1},\;I_{2},\;\ldots ,\;I_{n}\right\}$ denote the set of persistence intervals in the persistence diagrams, where $I_{i}=\left[b_{i},\;d_{i}\right]$ denotes the start and end time of the *i*-th persistence interval, p(i) denotes the probability that the duration interval $I_{i}$ occurs in the dataset. The persistence entropy PE(X) [19] is defined as:

(1) $$PE(X)=-\sum_{i=1}^{n}p(i)\log_{2}p(i)$$

Persistence entropy represents a measure of the complexity of persistent features. If the entropy value is high, it means persistence features are more complex; if the entropy value is low, it means the feature distribution is simpler.

Based on the persistence entropy, we define the Norm Entropy (NE(X)):

(2) $$NE(X)=\frac{PE(X)-\mu_{PE(X)}}{\sigma_{PE(X)}}$$

where *μ* is the mean of the entropy value and σ is the standard deviation. Equation (2) clearly describes the complexity of the topological features, meaning that it can describe the active nature of the nodes.

The NE(X) reflects the distribution of different topological features in the persistence diagram, when the NE(X) is high, it indicates that the topology of the dataset has a large diversity and complexity, which is the active; the NE(X) is in the middle of the dimensional general users; the NE(X) is low, it indicates that the topology of the dataset is relatively simple and consistent, which is the inactive.

After obtaining the NE(X) of each ego network, we input the 0-dimensional, 1-dimensional, and 2-dimensional NE(X) of each ego network as 3D feature vectors into the machine learning model. At the same time, we also tried to use only 1- and 2-dimensional NE(X) as inputs to the feature vectors for classification analysis. Finally, we compared the classification results of these two input methods.

## 4. Numerical Experiments

This chapter shows the classification results and the values of the evaluation metrics on three real social network datasets.

### 4.1. Datasets and Evaluation Indicators

In this paper, three real-world social network datasets from a network repository are selected, as shown in Table 1.

This paper uses three evaluation metrics to assess the classification results: Silhouette Coefficient: the Silhouette Coefficient ranges from [−1, 1], where a value higher than 0.5 is generally considered a relatively good clustering result. Calinski–Harabasz Index: a higher index value indicates better clustering results, with high intra-cluster similarity and significant inter-cluster differences. Davies–Bouldin Index: a lower index value signifies better clustering results, with low intra-cluster variance and high inter-cluster differences.

### 4.2. Classification Results

In this paper, after obtaining the ego network of each node in each of the three networks, the persistence homology of each ego network is computed separately and, after visualizing it as each persistence diagram, the persistence entropy of each persistence diagram is computed, and then NE(X) is computed and fed into three different machine learning active and inactive categories. This Section shows the classification results of three different datasets using the three machine learning methods. The classification results are then evaluated using three evaluation metrics and the results are shown in Table 2, Table 3 and Table 4 below.

As seen from the above table, in the Wiki-Vote network, the Silhouette Coefficients are all greater than 0.5, the Calinski–Harabasz index is very high, and the Davies–Bouldin index is a relatively low value, which implies that the data points are tightly clustered within their respective clusters and that different clusters are far apart from each other. This indicates that the classification results are good, and support vector machine and decision tree are better compared to k-means. In the Pages-Food network, the Silhouette Coefficients are all greater than 0.5, the Calinski–Harabasz index is higher and the Davies–Bouldin index is a relatively low value, which indicates that the classification results are good. In the Email network, the Silhouette Coefficients are greater than 0.5, the Calinski–Harabasz index is high and the Davies–Bouldin index is a relatively low value. Due to the relatively simple structures of the Wiki-Vote and Pages-Food networks, particularly in terms of link density and connection patterns between nodes, both SVM and decision trees are able to capture similar clustering patterns in these two networks. In contrast, the Email network is likely more complex, containing more noise and outliers, resulting in more pronounced differences in the clustering outcomes.

This shows that the proposed method is an effective method, and in order to optimize the results, in the following, we have compared the classification results for different dimensions with different classifications, respectively, and have obtained better results.

### 4.3. Comparison of Different Dimensions

When computing persistent homology, topological features on different dimensions can be obtained; in this Section, persistent homology is used in combination with k-means machine learning on three real social networks for classification comparison, and the results are shown in Table 5.

From the above table, it can be seen that no matter the network, choosing the combination of persistence features of one and two dimensions is better for classification; this is because when classifying users according to their activity level, users communicate with each other for the one-dimensional persistence features as well as higher two-dimensional persistence features, so choosing the combination of only these two dimensions is better than choosing the combination of three dimensions. This provides a new perspective for classifying users.

### 4.4. Comparison of Different Classifications According to Activity Level

In social networks, there are several types of regular users with different degrees of activity between active users and inactive zombie users. In this Section, we chose one and two dimensions combined high-dimensional NE(X) to divide the users into multiple categories starting from two categories on three real social networks for comparison, and the average degree of active users is calculated, as shown in Table 6.

From the above table, it can be seen that the average degree of active users, after dividing them into more than two categories, is greater than the average degree of active users after dividing into two categories in the three social networks because some of the users in the social networks have a small number of exchanges and are not zombie users. In the Email network, the lowest number of active users and the largest average degree of active users was after being classified into five classes, which means that classifying into five classes is the best; in the Pages-Food network, the largest average degree of active users was after being classified into six classes, which is the best classification; and in the Wiki-Vote network, the largest average degree of active users was after being classified into seven classes, which is the best classification. The average degree of active users after classifying users by degree ranking is calculated below for each network (the number of users in each class is the same as the number found by the method in this paper), as shown in Table 7.

From the above table, it can be seen that the average degree of this paper’s method is not the same as the classification according to the degree ranking, and the average degree of this paper’s method is greater for the active people, which indicates that this paper’s method is an effective method for classifying users in social networks.

## 5. Conclusions and Future Work

In this paper, we propose a new method for analyzing user activity in online social networks. First, an ego network is created for each user and the first two hops are selected as connecting edges between nodes. Then, we compute the persistent homology of the ego network and encode its topological features as network features. After obtaining the persistence entropy of each ego network, we computed the NE(X) value of each ego network and then input the zero-, one-, and two-dimensional NE(X) into the machine learning model as 3D feature vectors. Also, we tried to use only one- and two-dimensional NE(X) for classification. The results show that selecting high-dimensional topological features is more beneficial for classification than using all topological features. Compared with the degree ranking, the method in this paper classifies the actives with a greater average degree, and thus the classification is better. The specific classification categories vary depending on the network. The method in this paper provides a new perspective for user activity analysis.

In our future work, we will focus on two main directions:

1. Combining persistent entropy with semantic information to study user classification in social networks. This approach allows us to more accurately capture user behavior patterns and characteristics, thereby improving the accuracy of classification.
2. Applying persistent entropy to dynamic social networks to study changes in user classification at different time points. This method helps us better understand the evolution of social networks and provides more dynamic and timely classification results for users.

## Figures and Tables

**Table 1:** Social network datasets [22].

Network Name	Number of Nodes	Number of Edges
Wiki-Vote	889	2915
Pages-Food	620	2103
Email	1133	5452

**Table 2:** Evaluation classification results in the Wiki-Vote network.

Name	Silhouette Coefficient	Calinski–Harabasz	Davies–Bouldin
K-means	0.62	2048.43	0.59
SVM	0.65	2626.29	0.51
Decision tree	0.65	2626.29	0.51

**Table 3:** Evaluation classification results in the Pages-Food network.

Name	Silhouette Coefficient	Calinski–Harabasz	Davies–Bouldin
K-means	0.60	1430.02	0.61
SVM	0.58	1261.44	0.60
Decision tree	0.58	1261.44	0.60

**Table 4:** Evaluation classification results in the Email network.

Name	Silhouette Coefficient	Calinski–Harabasz	Davies–Bouldin
K-means	0.57	2317.8	0.62
SVM	0.60	2294.39	0.57
Decision tree	0.60	2724.35	0.54

**Table 5:** Classification results.

Network	Classifications	Active Users	Other Users	Silhouette Coefficient
Email (0, 1, and 2 dimensions)	2 classifications	531	602	0.57
	3 classifications	315	326,492	0.52
Email (1 and 2 dimensions)	2 classifications	480	653	**0.63**
	3 classifications	253	320,560	**0.62**
Pages-Food (0, 1, and 2 dimensions)	2 classifications	193	427	0.60
	3 classifications	115	228,277	0.58
Pages-Food (1 and 2 dimensions)	2 classifications	172	448	**0.74**
	3 classifications	90	113,417	**0.73**
Wiki-Vote (0, 1, and 2 dimensions)	2 classifications	326	563	0.62
	3 classifications	265	278,346	0.52
Wiki-Vote (1 and 2 dimensions)	2 classifications	321	568	**0.74**
	3 classifications	117	201,571	**0.74**

**Table 6:** The average degree of Active Users.

Network	Classification	Active Users	Other Users	Average Degree of Active Users
Email	2 categories	480	653	17.206251
	3 categories	253	320, 560	18.743083
	4 categories	251	533, 108, 241	18.832669
	**5 categories**	**133**	**533, 196, 111, 160**	**22.030075**
	6 categories	111	436, 155, 138, 196, 97	20.450451
Pages-Food	2 categories	172	448	14.227979
	3 categories	90	113, 417	19.677778
	4 categories	56	104, 417, 43	20.535714
	5 categories	53	325, 76, 43, 123	20.849057
	**6 categories**	**47**	**325, 55, 43, 92, 58**	**22.234043**
	7 categories	49	325, 53, 92, 23, 20, 58	21.571429
Wiki-Vote	2 categories	321	568	13.046729
	3 categories	117	201, 571	14.555556
	4 categories	103	127, 543, 116	17.398058
	5 categories	105	543, 79, 40, 122	17.190477
	6 categories	86	87, 46, 484, 113, 43	18.930233
	**7 categories**	**51**	**93, 484, 43, 83, 59, 76**	**24.705882**
	8 categories	52	86, 484, 40, 59, 73, 60, 35	24.480771

**Table 7:** Average degree of active users.

Network	Active Users	Average Degree of Active Users
Email	480	7.110416667
	**253**	**7.134387352**
	251	7.12749004
	133	5.932330827
	138	5.985507246
Pages-Food	172	5.831395349
	90	6.488888889
	56	6.946428571
	53	6.905660377
	**47**	**7.063829787**
	49	6.979591837
Wiki-Vote	321	5.327102804
	**117**	**5.333333333**
	103	5.194174757
	105	5.257142857
	86	4.930232558
	51	4.215686275
	52	4.25

## Data Availability

The data presented in this study are available on request from the corresponding author.
